# Controversies in Cavernous Malformation Management: A Comprehensive Review of Current Literature

**DOI:** 10.3390/jcm14238614

**Published:** 2025-12-04

**Authors:** Erika Carrassi, Edoardo Mazzucchi, Laura Raus, Mario Lecce, Laura Marucci, Alessia Farneti, Stefano Telera

**Affiliations:** 1 Neurosurgical Department, Santa Maria della Misericordia Hospital, 45100 Rovigo, Italy; 2 Neurosurgical Department, IRCCS Istituto Nazionale Tumori Regina Elena, 00144 Rome, Italy; 3 Radiotherapy Department, IRCCS Istituto Nazionale Tumori Regina Elena, 00144 Rome, Italy

**Keywords:** cavernous malformation, stereotactic radiosurgery, epilepsy, pregnancy

## Abstract

Intracranial cavernous malformations (CMs) are angiographically occult, slow-flow vascular lesions composed of dilated, mulberry-like capillary clusters lacking intervening brain parenchyma. CMs typically have a low annual hemorrhage risk and are often discovered incidentally. Most patients are asymptomatic or exhibit mild neurological symptoms at the time of diagnosis. Despite decades of investigation, the optimal management of CMs remains controversial. Key clinical dilemmas include identifying which lesions warrant active treatment and when, selecting the best therapeutic approach based on patient age and lesion location (eloquent vs. non-eloquent areas), and determining how to address the hemosiderin rim often found surrounding the malformation. Additional questions involve the role of radiosurgery and appropriate management strategies during pregnancy. This review critically evaluates current literature concerning the natural history and treatment strategies for CMs, emphasizing evidence-based approaches to these unresolved issues. By summarizing and interpreting recent findings, we aim to provide a concise yet comprehensive overview to support clinicians in tailoring patient-specific management plans for this complex neurovascular pathology.

## 1. Introduction

Cavernous malformations (CMs), also known as cavernous angiomas, are angiographically occult vascular anomalies consisting of irregular vascular channels lined by a single layer of endothelial cells lacking tight junctions, with no intervening nervous tissue between vessels. They represent 10–15% of all vascular malformations in the central nervous system (CNS) and are defined as slow-flow venous malformations according to the 2022 International Society for the Study of Vascular Anomalies (ISSVA) classification. In population-based studies, the incidence of CMs has been estimated between 0.15 and 0.56 per 100,000 persons [1,2,3,4].

Any site of the CNS may be involved. CMs are predominantly located in the supratentorial region (75%), followed by the brainstem (18%) and the basal ganglia/thalamus (8%). Multiple lesions occur in about 15–20% of cases, although this proportion may vary slightly between series. Incidental diagnosis in asymptomatic patients is observed in up to 60% of the cases [2,3]. Other less common sites include the pineal gland, middle cranial fossa, cerebellopontine angle, cavernous sinus, and optic nerve or chiasm. Localization at the level of the spinal cord is found in 5% of cases, most commonly as intramedullary or epidural lesions [2]. 

**Figure 1:**
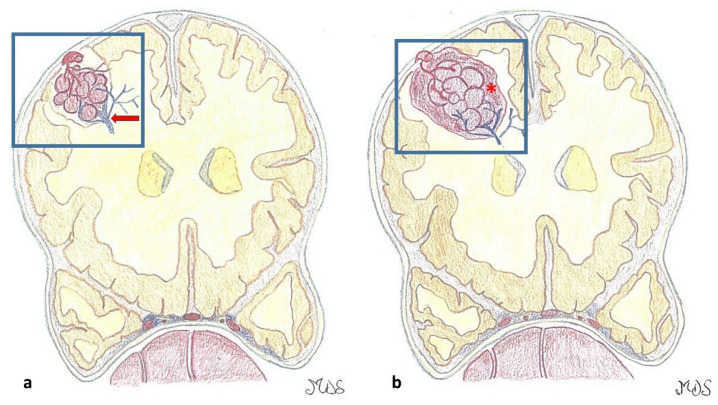
(**a**). Cavernous malformations (CMs) are round lesions with a brownish discoloration and an irregular, lobulated surface with a typical mulberry-like appearance, composed of a network of dilated capillaries without intervening neural, muscular, or elastic tissue. An associated developmental venous anomaly DVA (red arrow), possibly associated with venous congestion, is present in around one-third of the cases. (**b**). The capillaries, lined by a single layer of endothelial cells lacking tight junctions, are leaky and allow erythrocyte diapedesis, resulting in surrounding blood (hemosiderin), perilesional gliosis, and inflammation. On the contrary, larger hemorrhages (*) may be symptomatic according to their anatomic area.

**Figure 2:**
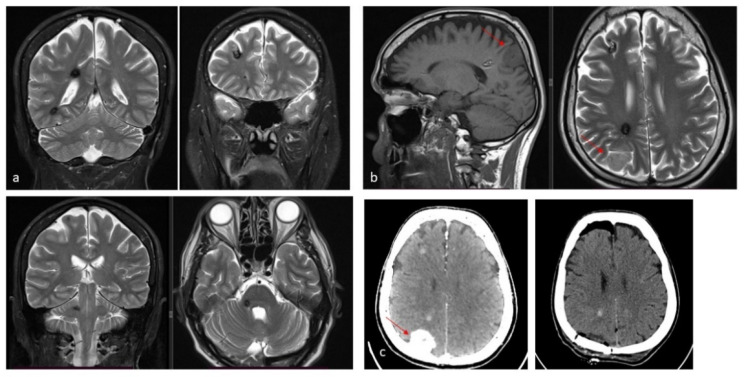
(**a**). Axial and coronal T2-weighted MRI of a 54-year-old patient affected by an incidentally discovered familial form of CM. Multiple asymptomatic CMs were observed supratentorially, infratentorially, and at the brainstem. (**b**). Sagittal T1-weighted and axial T2-weighted MRI showing a concomitant symptomatic parieto-occipital meningioma, which required surgery (red arrow). (**c**). Pre- and postoperative CT scan with contrast showing total removal of the meningioma (red arrow) and multiple hyperdense lesions corresponding to CMs.

A sporadic and an autosomal dominant familial form can be distinguished. About 50–70% of patients manifest the sporadic form of the disease. Typically, these patients present with a single lesion, often associated with a concomitant venous developmental abnormality (DVA). However, multiple CMs can occasionally occur in the sporadic form, usually clustered around the DVA. Recent high-field (7 Tesla) MRI studies further suggest that a DVA may be present in virtually all sporadic cases [5]. The contemporary occurrence of CMs and DVA is the most common mixed vascular malformation, with an incidence of 13–40% of cases. The pathophysiology is still uncertain, but they tend to coexist in the same territory. Three genes seem to be implicated in the etiopathogenesis of the familial form of the disease: KRIT1 (CCM1) on chromosome 7q21.2, MGC4607/malcavernin (CCM2) on chromosome 7p13, and PDCD10 (CCM3) on chromosome 3q26.1 [6]. The behavior of CMs is very similar in the two forms, except that in familial cases the lesions tend to be multiple [7]. The macroscopic appearance is that of a round lesion with a brownish discoloration and an irregular, lobulated surface resembling a blackberry, surrounded by a gliotic astrocytic halo and hemosiderin remnants secondary to recurrent intralesional microhemorrhages [8] (Figure 1 and Figure 2).

The clinical manifestations of CMs include seizures, headache, and focal neurological deficits. In most cases, hemorrhage from a CM is not life-threatening. With advances in neuroradiology, CMs are being detected more frequently, including in patients who are asymptomatic or only mildly symptomatic. This increased detection has improved our understanding of the natural history of the disease, but has also raised questions regarding the management and follow-up of incidentally discovered lesions [3].

On neuroimaging, CMs exhibit characteristic features. MRI is the modality of choice, with lesions typically demonstrating a ‘popcorn-like’ appearance due to mixed signal intensities from hemorrhage at various stages. A peripheral hypointense rim on susceptibility-weighted or T2-weighted sequences reflects hemosiderin deposition, which is a hallmark of these lesions. Unlike arteriovenous malformations, CMs lack large feeding arteries or draining veins on angiography, rendering them angiographically occult. Recognizing these imaging features is crucial for accurate diagnosis, management planning, and differentiation from other intracranial pathologies. The Zabramski classification of CMs was introduced in 1994 and, although not often used in clinical practice, remains the most popular classification in the scientific literature [9] (Table 1).

In a prospective study, Zabramski type I and II CMs were found to be associated with a significantly increased annual hemorrhage rate [10].

To date, the strategies available for treating CMs range from surgery to stereotactic radiosurgery (SRS) to simple observation. The treatment of these lesions is not particularly controversial when they are symptomatic and located in easily accessible, non-eloquent areas. On the other hand, the management of CMs located in eloquent areas remains highly debated. In these cases, surgical resection is generally recommended in cases of repeated hemorrhages, progressive neurological deficits, or drug-resistant epilepsy, with careful consideration of the risks associated with both the natural history of the disease and surgical intervention [11]. Another highly debated issue concerns the control of epilepsy (where present) and the removal of the haemosiderin ring. Here, the first distinction also concerns the location of the CM: if it is in a non-eloquent area, it is advisable to remove the haemosiderin ring as well. Conversely, in an eloquent area, this could cause neurological deficits and, therefore, the assessment remains case-dependent. Several studies have reported improved seizure control with early surgery and demonstrated a linear correlation between preoperative clinical status and postoperative outcomes [12]. Given this evidence and the well-established increased risk of rebleeding after the initial hemorrhage, a conservative “wait-and-see” approach may expose patients to the danger of irreversible neurological deterioration [13].

Therefore, the most crucial factor in choosing the best treatment for CMs, whether surgical, radiosurgical, or conservative, depends on the appropriate selection of patients and the evaluation of the risk of rebleeding. To date, this selection process is often influenced not only by objective factors but also by subjective aspects related to patient preferences and the surgeon’s experience. The influence of these subjective factors varies significantly, making it challenging to establish uniform recommendations [14]. Additional questions involve the pathophysiological role of hemosiderin rim circumventing CMs, the efficacy of SRS, the effects of surgery and SRS on epilepsy, and the appropriate management strategies of CMs during pregnancy.

## 2. Materials and Methods

This review critically evaluates the current literature (the last ten years 2015–2025), concerning the natural history and treatment strategies for CMs, emphasizing evidence-based approaches to these unresolved issues: (1) the risk of bleeding of CMs, (2) the indications of SRS, (3) the role of hemosiderin rim surrounding CMs and the effects of surgery and SRS on epilepsy and (4) the appropriate management strategies during pregnancy. By summarizing and interpreting recent findings, we aim to provide a concise yet comprehensive overview to support clinicians in tailoring patient-specific management plans for this complex neurovascular pathology.

## 3. Relevant Sections and Discussion

### 3.1. The Risk of Bleeding in CMs

The definition of a symptomatic intracerebral hemorrhage (ICH) due to a CM was standardized in 2008 as an “acute or subacute onset symptoms (headache, epileptic seizure, impaired consciousness, or new/worsened focal neurological deficits referable to the anatomic location of the CM) accompanied by radiological, pathological, surgical, or rarely only cerebrospinal fluid evidence of recent extralesional or intralesional hemorrhage”. On the contrary, the presence of a hemosiderin ring or the increase in diameter of the lesion alone could not be considered as a new hemorrhagic event [3,15,16].

Endothelial cells in hemorrhagic CMs frequently exhibit large intraluminal vesicles and absent or disorganized tight junctions. Thrombi, encapsulated hematomas, and granulation tissue are commonly observed. However, hemorrhage in CMs likely results from multiple contributing factors, and the precise pathogenic mechanisms remain largely unknown. For instance, according to recent reviews, the presence of a DVA might play a significant role not only in the development and growth of CMs but also in determining an increased hemorrhagic risk [17,18,19,20,21,22,23,24]. Moreover, the pathogenesis of recurrent bleeding may involve local overexpression of anticoagulant receptors such as thrombomodulin (TM) and Endothelial Protein C Receptor (EPCR). TM and EPCR are elevated in CM endothelial cells and plasma but absent in surrounding brain capillaries, potentially promoting localized thrombosis, hypoxia, and sinusoidal rupture, leading to recurrent hemorrhage and lesion expansion. Local differences in the distribution of these important regulators of blood coagulation may promote thrombosis in the dilated caverns, where blood flow is slow, or in an associated DVA, leading to hypoxia in the surrounding tissue and activating localized anticoagulant pathways. These can lead to sinusoidal ruptures, recurrent hemorrhages, and further expansion of the lesion [2,25].

**Figure 3:**
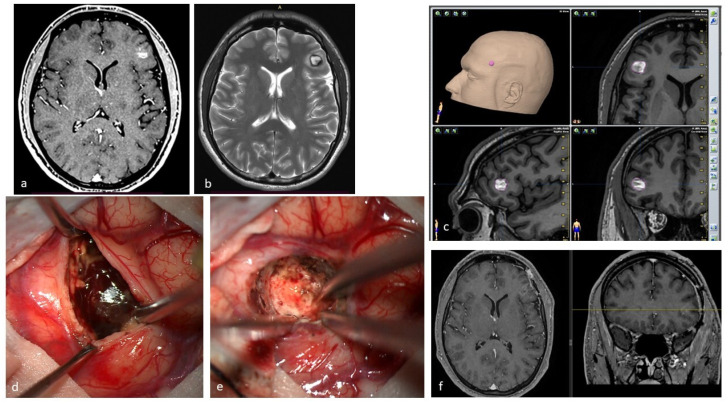
(**a**). Axial T1-weighted MRI with Gadolinium of a 27-year-old male affected by recurrent epileptic seizures, showing a frontal subcortical hyperdense lesion. (**b**). T2-weighted axial MRI showing a Type I CM according to Zabramski classification, reflecting subacute hematoma. The hypointense halo around the lesion corresponds to the hemosiderin ring. (**c**). Preoperative navigation planning with a T1-weighted MRI. (**d**). Intraoperative view showing the exposed mulberry-like CM. (**e**). Intraoperative view of the surgical field at the end of removal showing normal brain parenchyma without signs of the hemosiderin ring. (**f**). Postoperative axial and coronal T1-weighted MRI with Gadolinium showing total removal of the lesion.

The reported incidence of first hemorrhage in prospective studies ranges from 0.1 to 2.7 lesions per year [17,26,27]. According to the meta-analysis by Horne et al., the pooled annual risk of hemorrhage is approximately 2.4% for all CMs and 4.5% for previously hemorrhagic lesions [28]. Natural history studies suggest an annual hemorrhage risk of 2.3–4.1% for CMs, whereas surgical series report 2.7–6.8% annually before intervention [9,29,30,31]. Incidentally identified CMs have a lower first-hemorrhage risk (0.08% per person-year) [32,33]. A first hemorrhage may destabilize a CM, increasing the risk of subsequent bleeding, with rebleeding rates estimated between 4.5% and 34% per year. The cumulative incidence of rebleed is 56% at five years and 72% at ten years [26,27,29,30,31,32]. Kondziolka et al. reported hemorrhagic rates of 0.6% for incidentally discovered CMs, 4.5% after one hemorrhage, and 32% after more than one [33]. The most consistently identified risk factors for bleeding are brainstem location and prior hemorrhage. Other putative factors, identified in some, but not all studies, include female sex, the presence of a DVA or a DVA–CM complex, larger lesion size, and persistent or new T1 hyperintensity on MRI [17,18,19,20,21,22,23,24,28,30]. CMs located in the brainstem and thalamus/basal ganglia have higher risks of initial and recurrent hemorrhage (2.3–8.7% for first events, 12–60% for recurrences) and greater persistent morbidity after a single hemorrhage (40–60%), with cumulative disability increasing after subsequent events [28,30,32]. On the other hand, superficial CMs more frequently cause epileptic seizures after the initial hemorrhage, whereas focal neurological deficits are less common [26,28,30]. Rebleeding occurs in approximately 40% of CM remnants post-surgery, highlighting the need for early postoperative MRI within 72 h, with consideration for reoperation if substantial residual lesions are present (Figure 3). Rebleeding risk may be time-dependent, increasing for at least five years after the initial hemorrhage and declining thereafter, particularly in women [34]. This “temporal clustering” can affect the interpretation of treatment efficacy, especially following SRS. However, not all natural history studies have confirmed a reduction in risk after five years, and some report a persistently elevated or fluctuating hemorrhage rate over time [27,29]. Reported cumulative rebleeding rates are approximately 14% within the first year and 56% within five years [30,35,36].

### 3.2. Cavernous Malformation and Stereotactic Radiosurgery

Although surgery remains the mainstay of treatment for surgically accessible hemorrhagic and symptomatic CMs, the role of SRS in the management of high-risk, symptomatic cavernoma lesions has been recently reconsidered [37,38,39,40,41,42,43]. In the past decades, the choice of SRS was subjected to several criticisms due to the high incidence of reported complications and the unconfirmed reduction in hemorrhagic risk [28,32,44]. It should be emphasized that much of the available data originates from older studies, conducted at a time when stereotactic radiosurgery (SRS) was limited by suboptimal target delineation using CT or less conformal MRI techniques, and by the use of higher radiation doses, often similar to those employed for arteriovenous malformations. Currently, obtaining reliable data from literature remains challenging due to several methodological inconsistencies. Ideally, studies should (1) adopt a standardized definition of clinical hemorrhage associated with cavernous malformations (CMs), (2) distinguish between the first hemorrhage rate for treated lesions (per lesion-year) and the annual rebleeding rate prior to treatment, (3) report hemorrhage rates separately for the first two years post-treatment and for subsequent periods, and (4) differentiate outcomes based on lesion location (superficial vs. deep-seated), as well as the number of prior hemorrhagic events (0, 1, or multiple) [44,45,46,47,48].

However, recent studies have proposed SRS as a safe and effective treatment for CMs. This shift has primarily resulted from increased clinical experience with SRS, which has enabled a reduction in radiation dose protocols, as well as from technological advancements that have improved the precision of target localization [26]. Contemporary SRS techniques incorporate several key strategies: (1) prescription doses are typically maintained within the 12–15 Gy range; (2) high-resolution, conformal MRI is utilized to precisely delineate the target; (3) treatment is generally reserved for lesions without signs of recent hemorrhage (Zabramski Types II and III), with a minimum waiting period of 3 months following the last bleed; and (4) the hemosiderin ring is carefully spared due to its speculated radiosensitizing effects. Additionally, if a DVA is associated with the cavernous malformation, it should be preserved during treatment, as irradiation of the DVA has been linked to a higher incidence of complications [26,49]. Only in a small series of 30 patients affected by hemorrhagic solitary non-brainstem CMs, Chung et al. observed that higher doses, with a cut-off of >16 Gy, significantly prevented rebleeding, without causing a significantly higher incidence of symptomatic perifocal edema [50]. SRS is generally considered in cases of surgically inaccessible CMs in eloquent brain areas with repeated hemorrhages or when the patient has comorbidities precluding surgery or refuses the operation. Although intracranial hemorrhages are usually mild in severity, repeated hemorrhages may lead to significant disability or even death. Moreover, CMs anatomic location can impact SRS efficacy and affect patient selection. Given the higher rates of rebleeding and morbidity reported for brainstem CMs, some studies have suggested a possible role for SRS as an active treatment option in this setting [48,49,50,51,52,53,54]. A potential limitation of SRS is the lack of immediate elimination of bleeding risk during the latency period required for complete vascular obliteration. Furthermore, assessing treatment efficacy is challenging, as these lesions are angiographically occult and volumetric changes may not be reliably detected on imaging after SRS. At the same time, even volumetric regression could be only related to hematoma resorption and/or be part of the natural history of these lesions [28,48]. As such, the evaluation of treatment success used by most SRS series is mainly dictated by the clinical follow-up of the patients and the hemorrhage rates after treatment [55].

Another controversy stems from the absence of RCTs demonstrating a clear benefit of SRS over conservative surveillance, a comparison that is inherently challenging to evaluate due to the difficulty in organizing such studies. Actually, the available literature on this topic mainly consists of retrospective series with their inherent limitations. In 2024, Sandman et al. reported their retrospective analysis of 265 patients affected by CMs managed conservatively (25% with brainstem CMs). After a median follow-up of 58 months, the annual bleeding rate was 3.7% with cumulative 5-year and 10-year bleeding risk of 19% and 25%, respectively. Based on the findings that (1) approximately three-quarters of patients remained functionally independent at follow-up, (2) death due to CM-related hemorrhage was rare (<1%), and (3) only 1 in 20 conservatively managed patients required intervention during follow-up, the authors concluded that patients without symptomatic hemorrhage or without brainstem CMs may be more appropriately managed conservatively [56,57,58]. That said, a prospective randomized trial is underway: the CARE (Cavernomas: A Randomized Effectiveness) trial, a feasibility study in the UK and Ireland, is comparing medical management alone versus neurosurgery or SRS for symptomatic cavernomas. This trial is particularly relevant because it directly addresses the current evidence gap: no large RCT has yet shown a clear benefit of SRS (or surgery) versus observation, and CARE may generate high-level data to inform treatment decisions [59]. At the moment, given the persisting uncertainties in the natural history of CMs, the final decision on different treatment options should be made on an individual basis, taking into account the patient’s location, behavior, age, medical conditions, patient preferences, and the expertise of the surgeon and radiotherapist [26].

#### 3.2.1. Radiobiology of SRS Treatment for CMs

The radiobiological effects of SRS on CMs are not completely understood yet. The reduction in hemorrhagic risk induced by SRS is assumed to be related to a progressive hyalinization and thickening of the wall of the endothelium of pathological vessels leading to thrombosis and luminal closure of vascular channels, as observed after radiosurgical treatment in arteriovenous malformations. These changes are reported to start as early as 4 months and continue for 2 or more years after SRS [29,60]. It has also been proposed that the effects of radiation on angiographically occult feeders of CMs may play a role [21]. Indeed, the reduction in bleeding rate seems to become effective within a 2-year latency period after SRS, falling from 32 to 8.8% patient/year [26,33,61,62]. However, controversy persists, as patients with cerebral CMs may experience temporally clustered hemorrhagic events interspersed with hemorrhage-free intervals, thereby obscuring the distinction between the natural history of the disease and the effects of treatment [60]. Gamma radiation has also been shown to promote angiogenesis via multiple pathways, primarily through the upregulation of angiogenic factors such as vascular endothelial growth factor (VEGF), hypoxia-inducible factor 1 alpha (HIF-1α), and basic fibroblast growth factor (bFGF). Notably, high doses of radiation have been associated with VEGF overexpression, potentially leading to neovascularization and contributing to the increased hemorrhage rates observed in the early attempts at stereotactic radiosurgery (SRS) for the treatment of cerebral CMs [63,64]. Regarding the timeline of SRS-induced cerebral edema, Harat et al. reported that the most pronounced edematous effects typically occur around 6 months following treatment, with an average duration of 15 months, based on a cohort of 34 patients who underwent LINAC-based SRS for various indications [65]. In the study of Chung et al., the median time for post-SRS perifocal brain edema was 11 months. The median duration was 28.5 (range, 6–141) months [50]. Evidence from the literature supports the principle of excluding both the hemosiderin ring and the DVA from the treatment volume during SRS planning [28,52,66,67]. Lunsford et al. advocated that radiation dose to the hemosiderin ring around CMs may damage neighboring neural parenchyma through the release of vasoactive cytokines [68]. Multiple small isocenters, especially in eloquent areas, such as the brainstem or basal ganglia, may reduce these effects, as those proposed by Karaslaan et al. mean prescription dose and mean number of isocenters 12.6 Gy/3.54, 13.6 Gy/3.0, and 14.3 Gy/2.47 in the brainstem, basal ganglia/thalamus, and lobar region, respectively [21,69]. Lindquist et al. reported SRS results for patients with DVA and showed a high rate of brain edema after obliteration of the drainage vein in those patients [55]. Conformal treatment methods may be helpful in avoiding DVA obliteration, whenever possible. Karaslan et al. reported a temporary morbidity rate of 20.9% and a persistent morbidity rate of 3.5% in patients with cerebral CMs and associated visible DVAs following SRS, supporting the importance of DVA preservation during dose planning to minimize the risk of adverse radiation effects (AREs) [21].

#### 3.2.2. Treatment of Brainstem and Basal Ganglia CMs vs. Lobar Non-Eloquent CMs

Brainstem and basal ganglia CMs often follow a more aggressive course with higher hemorrhage risk, while surgical intervention carries substantial neurological risk [70,71]. The five-year risk of recurrent hemorrhage is estimated at 30.8% for brainstem CMs presenting with intracerebral hemorrhage or focal deficits, compared to 18.4% for CMs in other locations [28,30,72,73]. Surgical removal in these regions remains challenging even for experienced surgeons, with 10–14% persistent morbidity, 1.5–1.9% mortality, and gross total resection achieved in ~90% of cases. Residual lesions further increase risk, with up to 60% rebleeding rate, annual hemorrhage risk of 0.5–2%, and estimated mortality of 6% [30,74,75,76,77,78].

**Figure 4:**
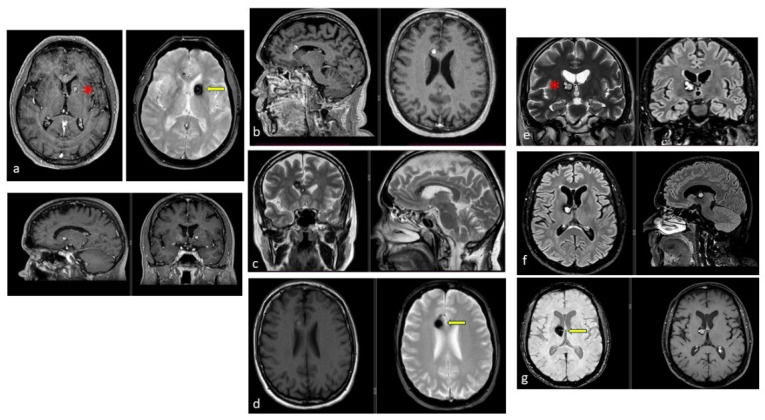
Examples of deep-seated asymptomatic CMs. (**a**). Axial, sagittal, and coronal T1-weighted MRI with gadolinium and axial GRE T2*-weighted MRI of the left caudate nuclei CM. (**b**). Sagittal and axial T1-weighted MRI with gadolinium. (**c**). Coronal and axial GRE T2*-weighted MRI and (**d**). Axial T1 and GRE T2*-weighted MRI, showing a CM of the corpus callosum. (**e**). Coronal T2-weighted and FLAIR MRI. (**f**) Axial and sagittal FLAIR MRI and (**g**). Axial GRE T2* and T1-weighted MRI showing a right thalamic CM. Note the “blooming artifact” on axial GRE T2*-weighted MRI due to pools of deoxygenated blood and deposits of hemosiderin and calcium (yellow arrows) and the classic “popcorn” appearance on FLAIR-weighted MRI with mixed signal intensity, expression of various blood breakdown products (red asterisk) of the CMs.

SRS, in contrast, appears to reduce hemorrhage while posing a lower complication risk. In long-term outcomes for brainstem CMs, Park et al. reported a 2.2% complication rate, with annual hemorrhage risk decreasing from 40.06% pre-SRS to 3–8% within two years and 1.48% at five years [37,52,79,80,81]. Similarly, Nagy et al. analyzed 113 patients treated with Gamma Knife SRS for 79 brainstem and 39 thalamic/basal ganglia CMs. In 41 high-risk lesions (>1 prior bleed), rebleed rates decreased from 30.5% per lesion-year pre-SRS to 15% in the first two years post-SRS, and further to 2.4% thereafter. These findings support SRS as a safe and effective option for CMs in eloquent areas, particularly in patients with multiple pre-treatment hemorrhages (Figure 4).

Although the benefit of treating single-bleed CMs is less certain, the morbidity associated with post-first hemorrhage events often exceeds that of SRS-related adverse effects (approximately 4%), thus potentially justifying active intervention [26,79]. Subsequent studies have supported early intervention, advocating for SRS within 3–6 months after the first hemorrhagic event, after clinical recovery and hematoma resolution [17,26,50,82,83,84].

Dayawansa et al., in a large multicenter study including 170 patients treated for brainstem CMs with a median margin dose of 12 Gy, observed a significant reduction of AHR from 14.8/100 CM-years (excluding first diagnostic bleed) to 2.3/100 CM-years. Overall, 42.3% improved clinically, 49.7% remained stable, and 8% worsened at a median follow-up of 3.4 years. No deaths occurred during the study. Notably, patients receiving a margin dose >13 Gy were at higher risk of post-SRS hemorrhage [46,85]. Two systematic reviews and meta-analyses have compared microsurgery to SRS for brainstem CMs, reporting that the composite outcome, including death, symptomatic ICH, and persistent disability, did not differ between the two treatments. However, persistent neurological deficits were significantly higher in the surgical group than in the SRS group. On the contrary, the number of patients with symptomatic ICH was significantly higher in the SRS group than in the surgical group [78,86]. Al Shalchy et al. recently performed a systematic review and meta-analysis including 45 studies and 3015 patients comparing conservative management vs. SRS vs. microsurgery for brainstem CMs. This study, albeit based mainly on retrospective studies with symptomatic patients, suggests that patients managed conservatively had the highest rebleeding rate (32.5%) and the lowest functional outcome rate (53.3%), while microsurgical resection resulted in lower recurrence, rebleeding, retreatment, and mortality compared to SRS and conservative management [87] (Table 2).

**Figure 5:**
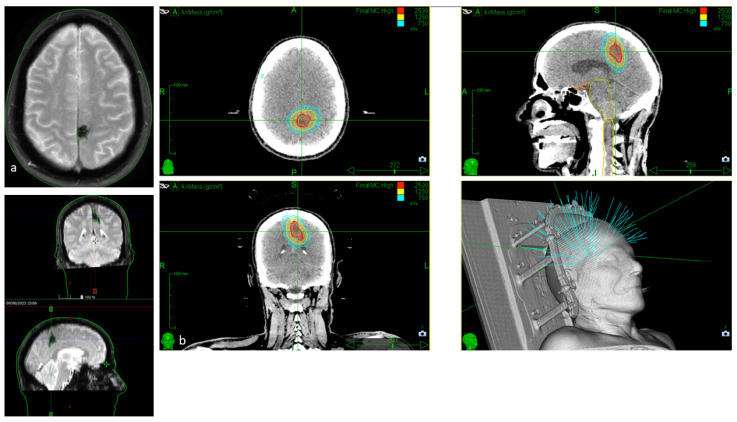
(**a**). Axial, coronal, and sagittal GRE T2*-weighted MRI of a 79-year-old patient with severe cardiologic comorbidities and a progressive motor and sensory deficit of the right leg and foot, showing a left parasagittal CM with typical blooming artifact. (**b**). SRS treatment planning of the same patient for whom surgery was considered too risky.

In summary, SRS is regarded as a valid and relatively safe therapeutic option for brainstem and eloquent CMs, particularly in symptomatic patients or those at high surgical risk. SRS has been shown to reduce the risk of rebleeding while maintaining an acceptable safety profile, although it does not replace microsurgical resection in surgically accessible and well-selected cases (Figure 5).

Due to generally favorable outcomes with surgery, superficial non-eloquent CMs are rarely treated with SRS, and thus, the literature on such cases is limited. Nagy et al. have explored the role of SRS in treating superficial CMs and the long-term effect on hemorrhage rates and epilepsy control in 96 patients. Median radiation dose was 15 Gy (range, 10–25 Gy), targeting a median lesion volume of 0.604 cm^3^. After a median follow-up of 7 years (range, 1–21 years) in lesions with multiple pre-treatment bleeds, the rebleed rate declined from 14.15% to 3.85% in the first two years and to 1.3% thereafter. Multivariate analysis identified younger age, deep lesion location, and multiple prior hemorrhages as predictors of post-treatment bleeding. Post-treatment hemorrhages or radiation-induced effects caused permanent deficits in 4.3% and 2% of patients, respectively. Epilepsy improved in 84.9% of patients after SRS, regardless of hemorrhage status or treatment timing. The authors concluded that SRS can be considered as a safe and effective alternative to surgery for selected patients, also with superficial CMs [88].

#### 3.2.3. Review of Recent Meta-Analysis

Given the absence of robust prospective RCTs, recent meta-analyses of published observational data have sought to substantiate the beneficial outcomes that SRS appears to offer in clinical practice for cerebral CMs (Table 3). Wen et al. (2019) [38] conducted a comprehensive meta-analysis to evaluate the efficacy and safety of SRS in the management of cerebral CMs.

Across the included studies, the annual hemorrhage rate significantly declined following treatment: from 7.2–39.5% pre-SRS to 1.22–12.3% within the first two years post-SRS, and further down to 1–3.6% beyond two years. Adverse effects were reported in 7.1% of patients, including headache and various neurological deficits, such as vertigo, facial palsy, dizziness, limb weakness, facial paresthesia, diplopia, dysarthria, cyst formation, and perilesional edema. Notably, larger lesion size and higher marginal doses were significantly correlated with a higher risk of post-treatment neurological deficits. The authors concluded that SRS appears to confer a significant reduction in annual hemorrhage rates, particularly in deep-seated and surgically inaccessible CMs, although a subset of patients may experience radiation-induced adverse effects [38].

Poorthuis et al. (2019) [89] conducted a systematic review of 30 cohort studies including 1576 patients (65% with infratentorial CMs) treated with SRS. Three studies directly compared SRS to surgery, and one included SRS, surgery, and conservative management, but none were randomized or blinded, leading to a high risk of performance and selection bias. Annual incidence rates for mortality, ICH, and focal neurological deficits were low (0.18%, 2.40%, and 0.71%, respectively), with a combined incidence of 3.63%, comparable to non-SRS patients. The authors concluded that randomized trials are needed to definitively assess SRS efficacy [89].

Kim et al. (2019) [90] conducted a systematic review and meta-analysis to provide more definitive evidence regarding the efficacy of SRS for brainstem CMs. The analysis included 576 patients from 14 retrospective studies. Following SRS, the annual hemorrhage rate significantly declined during the first two years and continued to decrease thereafter, from 23.35% to 3.2%. At the last follow-up, lesion volume had decreased in 47.3% of patients, remained stable in 49.4%, and increased in only 2.1%. Symptomatic AREs occurred in 42 patients (7.3%), with transient AREs observed in 24 patients (4.8%) and permanent AREs in 11 patients (2.2%). The use of a marginal dose ≤13 Gy was associated with a lower incidence of AREs. The authors concluded that SRS represents an effective therapeutic option for brainstem CMs, offering a favorable safety profile, particularly when lower marginal doses (approximately 13 Gy) are employed [90].

Bubenikova et al. conducted the largest meta-analysis comparing microsurgery, SRS, and conservative management for CMs, analyzing 98 studies with 8994 patients. Lobar, deep-seated, cerebellar, and brainstem CMs were considered separately due to differences in treatment approaches and outcomes. Most studies were retrospective, with only one RCT. Surgical intervention showed the lowest hemorrhage rates (3%), while conservative management had the highest case fatality (4%) and long-term morbidity (22%). Treatment efficacy was highest after surgery (97%) and lowest with observation (77%). SRS was particularly relevant for deep-seated, cerebellar, and brainstem CMs, where surgical access is limited. Initially, ICH predicted a higher post-SRS bleeding risk. Conservative management was associated with lower bleeding risk in lobar or cerebellar CMs, and higher risk in brainstem lesions, partly reflecting treatment selection bias. Overall, the analysis confirmed that surgery is highly effective in preventing hemorrhage, SRS is a valid alternative for surgically inaccessible or eloquent lesions, and observation remains reasonable for low-risk lesions without neurological deficits or seizures [28,30,91,92].

Shanker et al. retrospectively analyzed 39 patients undergoing SRS for symptomatic CMs and conducted a meta-analysis of 25 studies. They observed a significant reduction in annual hemorrhage rates from 52.1% pre-SRS to 12.3% post-SRS, while seizure incidence did not change significantly. Most of the hemorrhage risk reduction occurred within the first two years, supporting SRS as an effective option with low treatment-related toxicity [69].

Tos et al. performed a meta-analysis of 32 studies including 2672 patients treated with single-fraction SRS. The brainstem was the most frequent lesion location (36.9%), followed by lobar (30.6%), deep (basal ganglia/thalamus, 19.7%), and cerebellar (5.1%). SRS led to a 5.9-fold reduction in overall hemorrhage risk, 3.5-fold in the first two years, and 9.1-fold beyond two years. Volumetric outcomes showed lesion shrinkage in 46.9%, stability in 47.1%, and an increase in 6.7% of cases. AREs occurred in 8%, with permanent AREs in ~2%. Lower prescription doses (<13 Gy) were associated with fewer AREs [25].

For comparison, surgical resection of deep-seated CMs carries higher risks: Pandey et al. reported a 31.3% complication rate, and Harris et al., in a meta-analysis of 5089 patients, found mortality up to 4%, and morbidity 7–9% for deep lesions and 35–50% for brainstem CMs, respectively, supporting surgery to be reserved for symptomatic, accessible and non-eloquent lesions [2,74,93].

### 3.3. Cavernous Malformation and Epilepsy

Epilepsy is the most frequent presenting symptom of cerebral CMs, affecting approximately 25% of supratentorial cases [12]. The underlying mechanism is not fully understood, though the leading hypothesis implicates chronic microhemorrhages, which deposit hemoglobin and hemosiderin in the surrounding brain parenchyma [12,16,94,95,96,97,98,99,100]. The deposition of hemosiderin in the brain tissue surrounding the CMs contributes to producing the typical hypointense peripheral ring on MRI T1 and T2-weighted images and the “blooming” dark appearance of this vascular malformation in the MRI gradient recall echo (GRE) T2*-weighted and susceptibility-weighted imaging (SWI) [101,102,103]. This iron-rich byproduct can induce oxidative stress, neuronal excitotoxicity, and reactive gliosis, ultimately disrupting neurotransmission and promoting epileptogenesis. Based on this model, resection of the hemosiderin rim has been proposed to improve seizure outcomes [101,102,103,104,105,106,107,108,109,110]. While a meta-analysis by Englot et al. did not demonstrate an improvement in seizure outcomes, subsequent literature reviews found significant differences in the case of hemosiderin rim resection [95,96,97,98,99,100,101,111,112]. Current International League Against Epilepsy (ILAE) recommendations [12] highlight the need for prospective studies to clarify this issue. In the meantime, removal of a limited perilesional rim in non-eloquent regions may be reasonable, supported by both pathophysiological rationale and clinical data. In complex cases, such as those with extensive hemosiderin, multiple CCMs, or eloquent location, precise localization of the epileptogenic zone is essential. Even extended lesionectomy with electrocorticographic guidance has been proposed, particularly in cases of temporal lobe CM, especially when associated with hippocampal sclerosis [106,112,113]. Nevertheless, it is not recommended as a standard procedure [95,96,97,98,99,100,101,102].

According to the Alliance to Cure Cavernous Malformation recommendations [16,55], a first seizure in the absence of hemorrhage warrants medical therapy. Surgery may be considered if medications are poorly tolerated or if the patient wishes to discontinue them.

SRS has already been proposed as an alternative for microsurgery, not only to reduce the rate of hemorrhage, but also to treat epilepsy. Epileptic CCM lesions referred to for SRS are usually surgically inaccessible or situated in eloquent brain regions. Due to this selection bias, which reflects real-world clinical practice, it is difficult to compare surgical resection with SRS in terms of seizure control. Tos et al. in their meta-analysis reported a seizure-free proportion of 49.9% and a seizure-reduction proportion of 30.6%, for an overall proportion of clinical improvement of 80.5% [25], comparable to the 73% initially reported by Regis et al. [114] and slightly lower than the 85–87% demonstrated by Nagy et al. [66]. In a more recent multicenter cohort by Dumot et al. [46] from the International Radiosurgery Research Foundation, which included 109 patients treated with SRS for CM-related epilepsy across 11 institutions, 59.6% of patients achieved Engel class I or II. In the same study, seizure onset less than 1.5 years before SRS was associated with better control after SRS.

Both resection and SRS may contribute to seizure reduction by lowering hemorrhagic risk. While resection offers the advantage of hemosiderin removal and potentially greater seizure control, it carries a higher risk of morbidity and is more suitable for non-eloquent lesions. SRS, in contrast, may be preferred in eloquent areas or in patients unfit for surgery. Ultimately, treatment decisions should be individualized, considering clinical factors and patient preferences.

### 3.4. Cavernous Malformation and Pregnancy

Currently, there are no established guidelines for the management of women with CMs during pregnancy. Although case reports and small case series have suggested a potentially increased risk of hemorrhage during pregnancy or delivery, the available evidence remains inconclusive [115]. It has been hypothesized that elevated levels of progesterone and estrogen during pregnancy may upregulate the expression of vascular endothelial growth factor (VEGF) and basic fibroblast growth factor (bFGF), thereby promoting vascular instability within the malformation [116]. However, the presence of estrogen or progesterone receptors within CM tissue has not been demonstrated [117].

Two large retrospective case series [118,119] and one prospective study [120] have specifically evaluated the risk of CM hemorrhage during pregnancy compared to nonpregnant periods. These studies did not find statistically significant differences in bleeding rates, either in women who experienced CM hemorrhage following pregnancy (assuming lesion preexistence) or in those with a known CM diagnosis who became pregnant subsequently. Importantly, factors such as prior hemorrhage, brainstem location, and familial CMs were not associated with an increased risk of bleeding during pregnancy. As a result, prophylactic surgical resection of asymptomatic CMs solely to reduce pregnancy-related risk is not recommended [17]. However, as highlighted by Bektas et al., these studies have several important limitations: they included relatively small patient cohorts, were largely retrospective, and may be affected by selection bias and incomplete reporting. Additionally, differences in lesion location, prior clinical history, follow-up duration, and diagnostic methods introduce heterogeneity that may limit the generalizability of the findings. The authors also noted that rare peripartum hemorrhages could be underreported due to the limited sample size and variable monitoring, and that these studies may not capture long-term outcomes beyond pregnancy [121].

The optimal mode of delivery in women with CMs is also not well defined [122]. Cesarean section has been more frequently reported in case literature, presumably to avoid hemodynamic fluctuations and increased intracranial pressure associated with vaginal delivery and Valsalva maneuvers. Nevertheless, the low-flow characteristics of CMs make such a mechanism of bleeding less plausible. A retrospective study evaluating the risk of ICH during delivery in women with CMs or arteriovenous malformations found no increased risk to either the mother or the fetus [123]. Furthermore, two large CM series reported a total of 367 deliveries (including both vaginal and Cesarean) and 149 vaginal deliveries without a single case of peripartum hemorrhage [118,120]. To date, no scientific evidence supports a general recommendation for Cesarean delivery in women with CM. Therefore, the mode of delivery should be individualized based on obstetric indications and multidisciplinary clinical judgment [124].

It should also be mentioned that female hormone therapy (estrogen and/or progesterone), including oral contraceptives or hormone replacement, has been associated with a potentially increased risk of intracranial hemorrhage in women with CMs, particularly in those aged 10–44 years. As highlighted by Bektas et al. [121], these findings are based on observational studies and have important limitations, including small sample sizes, potential confounding factors, and incomplete adjustment for prior hemorrhage or lesion characteristics. These observations support the hypothesis that hemorrhage may be triggered by thrombus formation in dilated caverns or associated DVAs, and call for caution and further research regarding the use of female hormones in patients with CMs [125,126].

### 3.5. Conclusions

The management of CMs remains a subject of ongoing debate. Despite advancements in understanding their natural history and treatment strategies, several key challenges persist, particularly in patient selection (for any management approach, including observation, surgery, or SRS), treatment planning based on lesion location and age, and the management of hemosiderin-related complications. Microsurgical resection remains the treatment of choice in cases of recurrent hemorrhage, progressive neurological deficits, or drug-resistant epilepsy, especially for lesions in non-eloquent areas. Meanwhile, SRS is emerging as a safe and effective alternative for deep-seated, high-risk, or surgically inaccessible lesions. Further studies are needed to clarify the optimal management of CMs during pregnancy and to better understand the role of the hemosiderin rim in epilepsy pathogenesis.

In conclusion, the treatment of CMs requires a multidisciplinary and individualized approach, considering lesion characteristics, clinical presentation, patient preferences, and the expertise of both surgeons and radiation specialists. Prospective studies are essential to improve risk stratification and therapeutic decision-making, with the goal of optimizing clinical outcomes in this complex neurovascular condition.

## Figures and Tables

**Table 1:** Zabramski’s classification of CMs [9].

Lesion Type	MRI Signal	Pathologic Features	Images
Type I	T1: hyperintense core T2: hyper- or hypointense core with surrounding hypointense rim	Subacute hemorrhage surrounded by a rim of hemosiderin-stained macrophages and gliotic brain	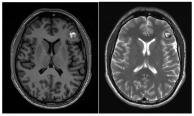
Type II	T1: reticulated mixed signal core T2: reticulated mixed signal core with surrounding hypointense rim resulting in the “popcorn” appearance GE: low signal rim with blooming	Loculated area of hemorrhage and thrombosis of varying age, surrounded by gliotic, hemosiderin-stained brain; in large lesions, areas of calcification may be seen	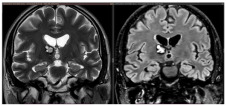
Type III	T1: iso- or hypointense T2: hypointense with a hypointense rim that magnifies the size of the lesion GE: hypointense with greater magnification than T2	Chronic resolved hemorrhage, with hemosiderin staining within and around the lesion	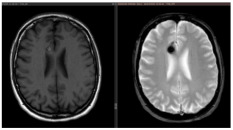
Type IV	T1: poorly seen or not visualized at all T2: poorly seen or not visualized at all GE: punctate hypointense lesions	Thought to be capillary telangiectasias	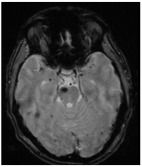

**Table 2:** Systematic Review and Meta-Analyses comparing microsurgery vs. SRS for brainstem CMs.

Authors	N° of Studies/Time Limit	Type of Studies	N° of Patients	Mean Age Years	Anatomic Location	Presenting Symptoms	Follow-Up	Results	Conclusions
**Gao,** **2021** [78]	Systematic Review and Meta-Analysis (1990–2019) Microsurgery vs. SRS	42 retrospective studies	2492 patients: 2122 microsurgery vs. 370 SRS	Microsurgery 37.2 (12–43) SRS 41.6 (37–44)	Brainstem (100%)	Cranial nerve dysfunction, Sensory disturbances, Motor palsy, hemiparesis, and headache	36 months (8–110)	Surgery was more commonly performed in symptomatic larger lesions located in the midbrain and pons, while SRS was preferentially selected in medulla CMs and in older patients. Mortality attributed to treatments, symptomatic ICH, and persistent disability did not differ between the microsurgery and SRS. PND was significantly higher in the surgical group than in the SRS group. On the contrary, the number of patients with symptomatic ICH was significantly higher in the SRS group than in the surgical group	Both microsurgery and SRS demonstrated great efficacy in reducing the rebleeding rate after treatment for brainstem CMs. Surgical removal of the symptomatic brain is generally favored. However, in specific cases, SRS is a valid alternative. Randomized trials are needed
**Fotakopoulos, 2021** [86]	Systematic Review and Meta-Analysis (last search 2020) comparing Microsurgery vs. SRS	6 retrospective studies	396 patients: 168 microsurgery vs. 228 SRS	range 33.3–43.7	Brainstem (100%)	-	-	No statistically significant difference or superiority between microsurgery and SRS of symptomatic brainstem CMs regarding PND after treatment, mortality rates, and reintervention	SRS for brainstem CMs seems to result in a marked reduction in the risk of rebleeding 2 years after treatment, but when compared with microsurgery, there was no remarkable difference. Microsurgery should be considered as the first-line management, particularly for lesions with progressive neurologic deficits, hemorrhage with mass effect, and small lesions. However, conservative management or SRS strategy may be considered for asymptomatic deep CMs and patients with severe comorbidities
**Al-Schalchy,** **2025** [87]	Systematic Review and Meta-Analysis (2001–2024) comparing Microsurgery vs. SRS vs. Conservative management	45 studies (44 retrospective cohort; 1 RCT)	3015 adult and pediatric patients: 83.9% microsurgery 10.7% RT or SRS 5.1% conservative management	10–58	Brainstem (100%) pons 62.4% midbrain 21% medulla oblongata 16.6%	Previous hemorrhage 82% Cranial nerve deficits 58.9% Hemiparesis 31.7% Ataxia 27.1% Incidental finding 0.7%	3–97 months (range)	No statistically significant reduction in rebleeding rate and mortality between conservative management and SRS. Significant reduction in rebleeding risk and mortality between microsurgery and conservative management Microsurgery was significantly associated with lower recurrence and mortality compared to SRS. Patients managed conservatively had the highest rebleeding rate and the lowest functional outcome rate	In patients with hemorrhagic or symptomatic brainstem CMs, microsurgical resection resulted in lower recurrence, rebleeding, retreatment, and mortality compared to SRS and conservative management. Radiosurgery may be suitable for selected patients with inaccessible or high-risk lesions. Randomized trials are needed

ARE. Adverse Radiation Effect. AHR Annual Hemorrhage Rate. SRS Stereotactic Radiosurgery RT. Radiotherapy RCT Randomized Controlled Trial. PND. Persistent Neurologic Deficit.

**Table 3:** Summary of the most recent meta-analysis reporting results of SRS treatment for CMs.

Authors	N° of Studies/ Time Limit	N° of Patients	Mean Age	Mean Size of CMs	Anatomic Location	Mean Marginal Dose	AHR Pre-SRS	AHR Post-SRS	Mean FU	Adverse Effect	Associated Factor to ARE	Conclusions
Wen, 2019 [38]	Meta-analysis 9 cohort studies (1 case–control) (2007–2017)	747	40 yrs	0.3–14.8 cm^3^	brainstem/basal ganglia/thalamus (81%)	11–15.8 Gy (range among studies)	7.2–39.5% (not including the first bleeding)	1.22–12.3% (first 2 yrs post-SRS) 1–3.6% (>2 yrs post-SRS) significant reduction	48 months (36–68)	7.1% (headache, FND, edema)		Patients with cerebral CMs, especially if deep-seated and surgically inaccessible, seem to benefit from SRS owing to a significant reduction in annual hemorrhage without differences between the first 2 years and 2 years after
Poorthuis, 2019 [89]	Meta-analysis 30 cohort studies (3 studies compared SRS vs. surgery; 1 study compared SRS vs. surgery vs. observation) (–2018)	1576	40 yrs	1.37 cm^3^ (0.6–1.86)	lobar (18%) basal ganglia and thalamus (13%) brainstem (61%)	15 Gy (13–16) (range among studies)		2.40% (symptomatic)	48 months (35–62)	0.71% (FND) 0.18% (mortality)		After SRS, the annual incidences of death, ICH, and FND are <5% and seem comparable to outcomes without SRS. A randomized trial of SRS for CMs is needed.
Kim, 2019 [90]	Meta-analysis 14 retrospective studies (2000–2018)	576	40 yrs	0.014–14.6 cm^3^	brainstem (100%)	11–15.8 Gy (range among studies)	23.4%	3.2%	6–228 months (range)	7.3% (2.2% permanent)	Marginal dose > 13 Gy	SRS using a relatively low marginal dose is a safe and effective treatment for brainstem CMs. Hemorrhage rate 2 years after SRS was significantly lower than that within 2 years after SRS.
Bubenikova, 2022 [30]	Meta-analysis 98 studies (1 single randomized study) (1990–2020)	8994	34.8 yrs	1.4 cm	lobar (23%) deep-seated (12%) brainstem (50%) cerellum (5%)			13.8% (symptomatic and asymptomatic)	50 months (23.6–112)	9% (long-term FND) 0.6% (mortality)	Deep-seated CMs Initial ICH	The efficacy of preventing hemorrhage was 97% in surgical, 86% in SRS, and 77% in conservative treatment. The lowest mortality (1%) was observed after SRS, and the highest persistent morbidity (22%) was observed in the conservatively treated group. SRS is a method of choice predominantly in poorly accessible CMs or those with a less aggressive nature.
Shanker, 2022 [69]	Meta-analysis 25 studies (1995–2021)	1758					24.9%	6.7% (first 2 yrs post SRS) 3.4% (>2 yrs post SRS) significant reduction	27–111.7 months (range)	12% (4% permanent)		SRS is effective and associated with a statistically significant eightfold reduction in rebleeding risk, particularly in the first 2 years following treatment.
Tos, 2024 [25]	Meta-analysis 32 studies (30 retrospective; 1 case–control; 1 prospective) (1998–2023)	2672	35–44 yrs	0.28–3.1 cm^3^	brainstem (36.9) lobar (30.6%) basal ganglia and thalamus (19.7%) cerebellum (5.1%)	11.6–25 Gy		Overall risk of AHR decreased by 5.9-fold (3.5-fold in the first 2 years post-SRS; 9.1-fold > 2 years post-SRS)	22–111 months (range)	8% (2% permanent) (6% if treated with < 13 Gy 9% if treated with > 13 Gy)	Marginal dose > 13 Gy	SRS is an effective intervention for CMs, significantly reducing hemorrhage rates and improving seizure outcome without differences based on marginal doses.

## Data Availability

Not applicable.

## Abbreviations

The following abbreviations are used in this manuscript:

CM	Cavernous Malformation
CNS	Central Nervous System
ISSVA	International Society for the Study of Vascular Anomalies (ISSVA)
DVA	Venous Developmental Abnormality
SRS	Stereotactic Radiosurgery
ICH	Intracerebral Hemorrhage
RCTs	Randomized Controlled Trials
AREs	Adverse Radiation Effects
